# Biochemical, photosynthetic and metabolomics insights of single and combined effects of salinity, heat, cold and drought in *Arabidopsis*

**DOI:** 10.1111/ppl.70062

**Published:** 2025-01-16

**Authors:** Elena Secomandi, Marco Armando De Gregorio, Alejandro Castro‐Cegrí, Luigi Lucini

**Affiliations:** ^1^ Department for Sustainable Food Process Università Cattolica del Sacro Cuore Piacenza Italy; ^2^ Department of Sciences, Technologies and Society Scuola Universitaria Superiore IUSS Pavia Pavia Italy; ^3^ Department of Plant Physiology, Facultad de Ciencias University of Granada Granada Spain; ^4^ Institute of Bioimaging and Complex Biological Systems (IBSBC) National Research Council (CNR) Milan Italy

## Abstract

Ensuring food security is one of the main challenges related to a growing global population under climate change conditions. The increasing soil salinity levels, drought, heatwaves, and late chilling severely threaten crops and often co‐occur in field conditions. This work aims to provide deeper insight into the impact of single vs. combined abiotic stresses at the growth, biochemical and photosynthetic levels in *Arabidopsis thaliana* (L.). Reduced QY max was recorded in salinity‐stressed plants, NPQ increased in heat and salinity single and combined stresses, and qP decreased in combined stresses. MDA and H_2_O_2_ content were consistently altered under all stress conditions, but higher values were recorded under salinity alone and in combination. Salinity alone and in stress combinations (especially with cold) provided a stronger hierarchical effect. Despite glycine and GABA osmolytes not significantly changing, proline highlighted the hierarchically stronger impact of salinity, while glycine‐betaine was decreased under drought combinations. Untargeted metabolomics pointed out distinct metabolic reprogramming triggered by the different stress conditions, alone or in combination. Pathway analysis revealed that abiotic stresses significantly affected hormones, amino acids and derivates, and secondary metabolites. Flavonoids accumulated under drought (alone and combined with heat and cold stresses), while N‐containing compounds decreased under all combined stresses. Looking at the interactions across the parameters investigated, antagonistic, additive, or synergistic effects could be observed depending on the biochemical process considered. Notwithstanding, these results contribute to delving into the impact of various stress combinations, hierarchically highlighting the stress‐specific effects and pointing out different combinations.

## 1 INTRODUCTION

Abiotic stresses, such as drought, heat, salinity, and cold, are responsible for a decline in crop yield, both qualitatively and quantitatively (Bashir et al., 2019), and represent worldwide limiting conditions (Cramer et al., 2011). Besides showing differences as a function of stress type, stress level and plant genotype, abiotic stresses can reduce yields by more than 50%, on average (Boyer, 1982; Vogel et al., 2019). Consequently, these stresses are a major threat to food production, becoming even more relevant in an ever‐growing human population under climate change. Indeed, climate change exacerbates exposure to abiotic stresses, with extreme temperatures, increased drought, or soil salinity accumulation as the most apparent threats.

The biology of plants' response to individual abiotic stresses has been extensively studied over the past decades, with several stress‐specific modulations (including acclimation) being elucidated at different biochemical and ecophysiology levels. Nonetheless, as reviewed by (Lasky et al., 2023), deeper information about the molecular basis of stress adaptation is still needed. Even more importantly, plants experience a combination of abiotic stresses under realistic field conditions rather than single stresses (Moffat, 2002; Mittler, 2006). Recent literature has pointed out that the response to combined stresses cannot simply be extrapolated from plant response to each individual stress (Pandey et al., 2015; Rizhsky et al., 2004; Suzuki et al., 2005). This suggests that a knowledge gap may exist between the information on plant impact provided by single stresses applied individually compared to multiple stress conditions (Mahalingam, 2015; Suzuki et al., 2014), representing more feasible and realistic field conditions.

Some common biochemical modulations can be observed across abiotic stresses, like the accumulation of osmolytes under drought, salinity, and chilling (Chinnusamy, 2003). On the other hand, plant response to a threat is specific and tailored to environmental stress conditions. A good example of this concept comes from reactive oxygen species (ROS), generally associated with most abiotic and biotic stressors but with significant differences in ROS‐gene expression patterns observed among different stresses (Mittler, 2002; Mittler et al., 2004). Combined stresses can be either additive or antagonistic to plants (Rillig et al., 2021). For example, conflicting responses are observed when plants open stomata during heat stress to cool leaves by transpiration, thus being more sensitive to co‐occurring drought stress. On the contrary, heat stress can increase tolerance to salinity by inhibiting the uptake of Na^+^ ions, promoting their accumulation in roots rather than in shoots (Rivero et al., 2014). These few examples make clear that plants' acclimation to co‐occurring abiotic stresses requires a combination of responses to individual stress conditions. Moreover, tailored responses are required to fine‐tune molecular processes accounting for the aspects arising from stress combination. Drawing upon the still limited information available in the literature on simultaneously occurring abiotic stresses, Mittler has developed the “stress matrix” where both positive and negative interactions have been proposed and postulated that stress combination should be regarded as a distinct state of abiotic stress (Mittler, 2006).

On these bases, our work aimed at investigating the combination of co‐occurring abiotic stress, using the model plant *Arabidopsis thaliana* L. and applying a combination of biochemical, photosynthetic and metabolomic analyses. The automated, high‐throughput phenotyping system monitored plant responses to stresses by evaluating plant growth, leaf shapes, and photosynthetic traits. In addition, the biochemical assays and targeted metabolomic of well‐recognized stress markers, together with untargeted metabolomics coupled with multivariate statistics and pathway analysis, aimed at unravelling the additive or antagonistic effects between drought, heat, chilling, and salinity.

## 2 MATERIALS AND METHODS

### 2.1 Plant material, growing conditions, and stress treatments

Plants of *Arabidopsis thaliana* L., accession Columbia‐0 (Col‐0) were grown from March to April 2023 at the facilities of Università Cattolica del Sacro Cuore (Piacenza, Italy). Seeds were stratified in distilled water and kept for 72 h at 4°C in dark conditions to synchronize the germination. Pots (6 × 6 × 9.5 cm) were prepared with 130 g of soil:perlite (1:2) mixture and watered one day before sowing up to maximum soil water holding capacity. Then, 5 seeds were sown per pot to be thinned after germination, leaving one seedling per pot. The growth chamber supplied by Ambralight (Ambra Elettronica, Bolzano Vicentino, Italy) was set to 20 ± 2°C, 8/16 h light/dark photoperiod, and 250 μmol m^−2^ s^−1^ photosynthetic photon flux density (PPFD). The seedlings of *A. thaliana* were watered every other day until reaching the 3.7 stage, 38 Days After Sowing (DAS) (Boyes et al., 2001). Plants were then randomly divided into nine groups, each corresponding to a different treatment, with four biological replicates per treatment.

The following treatments were applied: CNTR (Control, unstressed plants), H (Heat), D (Drought), C (Cold), S (Salinity), D × H (Drought x Heat), D × C (Drought × Cold), SxH (Salinity × Heat), S × C (Salinity × Cold). Drought was applied by blocking irrigation until they reached a relative water content (RWC) of around 70% (9 days). Salinity stress was applied by watering plants with a 100 mM NaCl solution daily until full water holding capacity for 9 days. Cold stress was induced by keeping the plants at 4°C for 16 h (dark period). To avoid heat shock, heat stress was applied in two steps: temperature was raised to 26°C for 14 h and then to 30°C for 6 h. Both the cold and heat stress were applied the day before the sampling. Other combinations were excluded in the experimental set up based on theoretical inconsistency (e.g., Cold × Heat) and technical incompatibility based on in‐house pre‐experiments (e.g., Drought × Salinity). At the end of the experiments, corresponding to 47 DAS, leaves collected for metabolomics and biochemical assays were immediately snap‐frozen to quench metabolism, powered in liquid nitrogen and stored at −80°C.

### 2.2 RGB imaging and Chlorophyll Fluorescence phenotyping

To investigate the effect of single and combined abiotic stress treatments, plants underwent high‐throughput phenotyping for RGB and chlorophyll fluorescence kinetics (ChlF) traits using the PlantScreen™ System (Photon System Instruments). The measurements were conducted starting before the treatment application at 38 and then at 40, 43, 44, 47 DAS (corresponding to T0, T1, T2, T3, T4 respectively). The PlantScreen™ Analyzer software (PSI, Czech Republic) was used to automatically process the raw data (Pixel count and fluorescence intensity) according to Awlia et al., 2016.

RGB images of 5 × 4 plants per tray were captured using an RGB2 top view camera (GigE PSI RGB, 1.4 Mega‐pixels with 1 / 2.3″ CMOS SENSOR). Light conditions, plant position and camera settings were fixed throughout the experiment. Each round of measurements included an initial 15 min dark adaptation period inside the acclimation chamber.

Chlorophyll fluorescence was acquired using the FluorCam FC‐800MF pulse amplitude modulated (PAM) system (PSI). Three types of light sources were used as part of the ChlF imaging station: (1) PAM short‐duration measuring flashes (620 nm), (2) cool‐white (6500 K) actinic lights with maximum irradiance 1860 μmol m^−2^ s^−1,^ and (3) saturating cool‐white light with maximum irradiance 6300 μmol m^−2^ s^−1^. Specifically, to quantify the rate of photosynthesis at different photon irradiances, an optimization of the Light Curve‐Act protocol was applied (Henley, 1993; Rascher et al., 2000), given its suitability to provide information on chlorophyll performances under stress (Brestic & Zivcak, 2013). A 5 s flash of light was applied to measure the minimum fluorescence, followed by a saturation pulse of 800 ms (with an irradiance of 1300 μmol m^−2^ s^−1^) to determine the maximum fluorescence in the dark‐adapted state. Next, 60 s intervals of cool‐white actinic light were applied at 115, 220, 325, and 430 μmol m^−2^ s^−1^ corresponding to L1, L2, L3, and L4, respectively. A saturation pulse was applied at the end of the period of actinic light to acquire the maximal fluorescence in the light‐adapted state. The ChlF signal measured just before the saturation pulse was taken as the steady‐state fluorescence value in the light‐adapted state. Fluorescence images were captured by a CCD camera at 16‐bit resolution in 1360 × 1024 pixels of CCD chip by FluorCam software included maximum PSII quantum yields (F_v_/F_m_), PSII quantum yield of light‐adapted plants (F_v_′/F_m_′), coefficient of photochemical quenching (qP) and non‐photochemical quenching (NPQ) in steady state.

The MorphoAnalyser software (version 1.0.9.6) was used to elaborate the acquired images and assess plant growth. The compactness and roundness of leaves were assessed, and the total number of pixels was successively converted to mm^2^ to calculate leaf projected areas.

### 2.3 Lipid peroxidation

Lipid peroxidation was determined as malondialdehyde (MDA) content by the TBARS assay (Heath and Packer, 1968; Castro‐Cegrí et al., 2023a) with minor modifications. 0.1 g of fresh leaves were extracted for each plant (4 plants per treatment) using 1.5 mL 20% trichloroacetic acid (TCA) (w v^−1^) and 0.3 mL 4% butylated hydroxytoluene (BHT) (w v^−1^). The homogenate was centrifuged twice for 10 min at 7142 *g* at 4°C. 0.25 mL of supernatant was mixed with 0.75 mL of 0.5% thiobarbituric acid (TBA) (w v^−1^). Three technical replicates were prepared for each sample. The mixture was incubated for 30 min at 94°C, then the reaction was stopped in ice for 10 min. The absorbance of the supernatant was then measured at 532 and 600 nm. Results were calculated using a calibration curve and expressed as μg of MDA per kg of fresh weight.

### 2.4 Hydrogen peroxide content

The content of hydrogen peroxide (H_2_O_2_) was determined as in (Alexieva et al., 2001) with minor modifications. 0.4 g of fresh leaves material was homogenized in 1.5 mL of 0.1% (w v^−1^) trichloroacetic acid and centrifuged at 4°C and 7142 *g* for 15 min. The reaction mixture comprised 0.25 mL of supernatant, 0.25 mL of 0.1 M potassium phosphate buffer pH = 7 and 1 mL of 1 M KI. Samples were incubated for 1 h in the dark at room temperature, and absorbance was measured at 390 nm. Results were calculated using a calibration curve and expressed as μg of H_2_O_2_ per kg of fresh weight. For each treatment, four biological replicates in three technical replications were carried out.

### 2.5 Electrolyte leakage

Electrolyte leakage was determined following the method proposed by (Castro‐Cegrí et al., 2023b). Each replicate consisted of four leaves of similar size (1 × 3 cm on average), and four replicates were measured per treatment. Leaves were rinsed with 50 mL of deionized water thrice for 3 min. After being incubated for 30 min and hand‐shaken in 30 mL of deionized water, this solution was measured for initial conductivity (Ci) at room temperature using a conductometer. Total conductivity (Ct) was then determined after boiling the flasks for 10 min and cooling at room temperature. The electrolyte leakage was expressed as a percentage of total conductivity: % electrolyte leakage = (Ci*100)/Ct.

### 2.6 Untargeted Metabolomic analysis

An accurate amount (0.2 g) of each sample was extracted using an ultrasonic bath (ArgoLab DU‐32) for 15 min at maximum power in 2 mL of 80% methanol (MeOH, purity ≥99.8%, Sigma‐Aldrich) solution with 0.1% (v v^−1^) formic acid (purity ≥95%, Sigma‐Aldrich). Samples were then centrifuged at 7142 *g* for 15 min at 4°C (Eppendorf 5430R), and 1 mL of the resulting supernatant was transferred in a vial using a 0.22 μm regenerate cellulose filter. Four independent replicates were analyzed for each treatment condition, with two technical replicates per sample. Quality Controls (QC) were prepared by mixing 20 μm of each extract and were randomly injected throughout the chromatographic sequence to avoid analytical bias.

The phytochemical profile was evaluated by ultra‐high‐pressure liquid chromatography equipped with a binary pump and a Dual Electrospray Jetstream ionization source, coupled to quadruple time of flight mass spectrometry (1290 UHPLC / 6550 iFunnel QTOF‐MS from Agilent Technologies) as previously reported by Salehi et al., 2023. Reverse phase chromatography was applied for separation using a water‐acetonitrile gradient elution from 6 to 94% of acetonitrile in 33 min, a flow rate of 0.2 mL min^−1^ and an Agilent Zorbax Eclipse Plus C18 analytical column (15 cm × 2.1 mm, 1.7 μm particle size). The mass spectrometer acquired data in positive SCAN mode in the 100–1200 m/z range. Blank samples were injected at the beginning and the end of each randomized sequence run. Moreover, QC samples were analyzed within each sequence every 9 samples in data‐dependent MS/MS mode (8 precursors per cycle, 1 Hz, 50–1200 m/z, positive polarity, active exclusion after 2 spectra), at 10, 20, and 40 eV collision energies.

Raw data were annotated by Profinder B10.0 (Agilent Technologies), applying the “find‐by‐formula” algorithm based on monoisotopic mass (5‐ppm tolerance for mass accuracy), isotope spacing and ratio. Compound annotation was carried out against the PlantCyc database 9.6 (Hawkins et al., 2021) following mass and retention time alignment of deconvoluted features. Level 2 of identification (i.e., putatively annotated compounds, COSMOS standards in metabolomics) was achieved (Salek et al., 2013). Data filtering was finally applied, and compounds not detected in at least 75% of the replications within at least one group were discarded.

### 2.7 Targeted analysis for osmolyte quantification

A targeted approach was used to quantify the osmolyte stress markers proline, glycine, glycine‐betaine, and γ‐aminobutyric acid (GABA). To this aim, a Vanquish ultra‐high‐pressure liquid chromatography coupled to a Q‐Exactive HF Hybrid Quadrupole‐Orbitrap mass spectrometer through a HESI‐II probe (Thermo Scientific) was used. The chromatographic and MS conditions were based on Khan et al., 2017. Briefly, a volume of 6 μL of the same extract used for untargeted metabolomics was injected. The separation was made by a water‐acetonitrile gradient elution of 50% in 12 min with a flow rate of 0.2 mL min^−1^ at 50°C using an Acquarity PREMIER Peptide CSH C18 analytical column (130A 1.7 μm, 2.1 × 150 mm, 1pk^−1^). The mass spectrometer acquired data in FULL SCAN mode in the 50–250 m/z range, with a nominal resolution at 70,000 full of at half maximum (FWHM) and in positive polarity. The Orbitrap mass analyzer operated in positive mode (ESI+) for both MS and MS/MS acquisition with nitrogen as both sheath gas (40 L min^−1^) and auxiliary gas (20 L min^−1^ and 50°C). The spray voltage was 3.5 kV with a capillary temperature of 250°C and an S‐lens RF level of 50. XCalibur 4.1.31.9 (Thermo Fisher Scientific Inc.) software was used for data acquisition and processing. Absolute quantification was achieved against calibration curves built with pure reference standards for proline, glycine, betaine, and GABA (all from Sigma‐Aldrich; purity >98%). Monoisotopic accurate mass, MS/MS spectral fragmentation, and retention time were used for identification (Table S1). Four biological replicates were analyzed for each treatment, and results were expressed as μmol g^−1^ FW apart from betaine data expressed as nmol g^−1^ FW.

### 2.8 Statistical analysis

One‐way ANOVA with Duncan post‐hoc test (*p* < 0.05) was carried out for the analysis of morpho‐physiological and photosynthetic data, biochemical assays and osmolytes quantification using SPSS 28 (IBM). Pearson's correlation analysis and univariate two‐way ANOVA for each stress combination were performed with the same software.

Metabolomics data were elaborated with the Mass Profiler Professional B15.1 software tool (Agilent Technologies) (Benjamin et al., 2019). Compound abundance was log2‐transformed, normalized at the 75th percentile and baselined against the median of all samples. According to their metabolic profile, the similarities and/or differences among samples were reported through unsupervised hierarchical cluster analysis (HCA ‐ Euclidean distance, Ward's linkage) based on fold change (FC) heat map. One‐way analysis of variance (ANOVA) was performed to detect the statistically significant compounds among treatments, setting a significance level of *p* < 0.05 (Tukey's post hoc test; Bonferroni multiple testing correction). Datasets were separately imported in SIMCA 17 (Umetrics, Malmo, Sweden) to perform supervised orthogonal projection to latent structures discriminant analysis (OPLS‐DA) multivariate modelling. The obtained model was successively cross‐validated (CV‐ANOVA; *p* < 0.05), inspected for outliers (Hotelling's T2), and the model's goodness parameters (goodness‐of‐fit R^2^Y and goodness‐of‐prediction Q^2^Y) were checked. Model overfitting was excluded by permutation testing (*n* = 100). Finally, the Variable Importance in Projection (VIP) analysis was used to select the metabolites having the highest discriminant potential score (VIP >1.2). Statistically significant (*p* < 0.05 in at least one of the treatments) VIP compounds were selected for biochemical interpretations in Pathway Tools Omics Dashboard (version 27.0) (Plant Metabolic Network, www.plantcyc.org, accessed on November 24th, 2023) (Paley & Karp, 2024).

## 3 RESULTS

### 3.1 Phenotyping and photosynthetic traits

**FIGURE 1:**
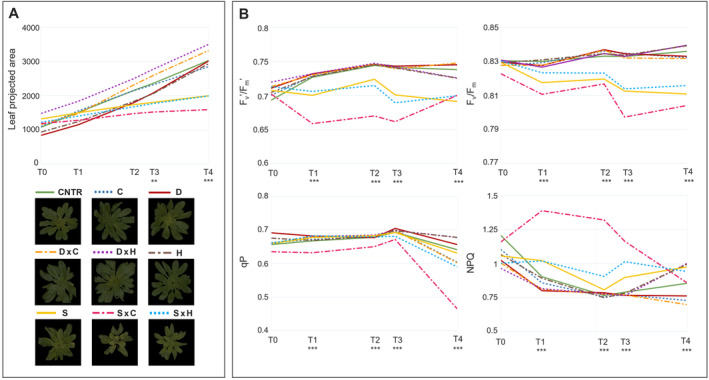
(A) Leaf Projected Area of *Arabidopsis thaliana* under control, single and combined abiotic stresses and RGB images of plants at T4 (B) Maximum quantum yield of PSII in dark‐adapted (F_v_/F_m_) and light adapted (F_v_′/F_m_′) leaves, non‐photochemical quenching (NPQ) and coefficient of photochemical quenching in steady state (qP) of *A. thaliana* plants under control, single and combined stress conditions. All data were acquired from 4 different biological replicates with the PlantScreen System; T0, T1, T2, T3, T4 correspond to 38, 40, 43, 44, 47 DAS. CNTR = Control, unstressed plants; H=Heat; C=Cold; D=Drought; S=Salinity; D×C=Drought × Cold; D×H=Drought × Heat; S×C=Salinity × Cold; S×H=Salinity × Heat. Significant differences between control and stress treatments are indicated with ** and *** for *p* < 0.01 and 0.001, respectively.

Phenotyping allowed the non‐destructive monitoring of plant growth throughout the entire experimental period. Data regarding the plant area showed no significant differences in the first part of the experiment, while from T3, corresponding to the 6th day of salinity, stress treatment reduced plant growth, and all the salt‐stressed plants were significantly different from the non‐salinity stressed ones (Figure 1A; Table S2). However, heat and cold stress applications had no impact on the plants' area, likely because of the limited duration of the stress. Similarly, drought stress did not affect the digital area of plants.

Other morphological parameters, including compactness and roundness, have been further addressed. In the late phase, significant differences in roundness could be observed in SxH‐treated plants while compactness never differed in the T0‐T4 measurements (Table S2).

To determine the physiological status of *Arabidopsis thaliana* plants under single and combined stress conditions, an automated chlorophyll fluorescence imaging was set based on the light curve protocol. The basic ChlF parameters were derived from the measured fluorescence transient states (e.g., F_0_, F_0_′, F_m_, F_m_′, F_t_, and F_v_) and then used to calculate the quenching coefficients (e.g., qP, NPQ) and other plant photosynthetic performance parameters (e.g., F_v_/F_m_, F_v_′/F_m_′). The maximum quantum yield of PSII photochemistry in the dark‐adapted (F_v_/F_m_) and the light‐adapted (F_v_′/F_m_′) states, the coefficient of photochemical quenching that estimates the fraction of open PSII reaction centers (qP), the steady‐state non‐photosynthetic quenching measuring heat dissipation (NPQ) for days 0, 2, 5, 6, and 9 of the stress‐application periods are showed in Figure 1B. The highest actinic photon irradiance (L4) was chosen to assess the photosynthetic activity variation as it provided the most discriminative power for evaluating abiotic stresses impact on ChlF parameters.

The analysis of NPQ and qP parameters allowed us to highlight interesting and diverse responses in the photosynthetic activity amid treatments. Upon exposure to salt stress, increased NPQ values were observed at varying degrees in salt‐treated plants starting from T1. At T4, after the temperature variation inductions, a strong increase could be recorded in heat‐treated plants (Figure 1B; Table S3). Regarding qP, a decrease was observed in the salt‐induced over time and in the cold‐treated plants at T4. Overall, considering the longer salt stress applications, this resulted in a rapid and substantial increase in non‐photochemical processes (i.e., the dissipation of heat in the PSII antennae), which correlates with reduced PSII quantum efficiency and photochemical quenching under stress (Figure 1B; Table S3). However, temperature induction resulted in a variation of NPQ and qP, respectively, for heat and cold stress, without impacting maximum quantum yield (F_v_/F_m_) values, likely due to the short time application. On the contrary, a progressive decline of F_v_/F_m_ was recorded in plants under salinity starting from T1 (Figure. 1B; Table S3). No significant change in the photosynthetic efficiency was observed in drought‐stressed plants during the 9 days of stress, suggesting no damage to PSII throughout the stress treatment. Comparable results were recorded for F_v_’/F_m_′, which did not change between control and stressed plants, except for salt‐treated samples.

### 3.2 Effect of combined stresses on oxidative stress markers

Abiotic stresses can increase reactive oxygen species, such as hydrogen peroxide (H_2_O_2_). As shown in Table 1, salinity and its combinations, as well as drought and its combination with heat, significantly increased the accumulation of H_2_O_2_ compared to the control, with the combination of salinity and cold stress being the treatment triggering the highest accumulation of this compound. Comparing the results obtained from MDA content, as an indicator of lipid peroxidation, all the stresses induced MDA accumulation, compared to control (Table 1). Furthermore, the salinity stress and its combinations showed the highest induction of this parameter than the other stresses, particularly for SxC, where an accumulation of 388% was recorded compared to the control (Table 1). The interaction between H_2_O_2_ and lipid peroxidation was evaluated through linear correlation analysis, showing a Pearson's correlation factor of 0.915 (*p* < 0.001) between the two stress markers. When analysing membrane stability, salinity was consistently the stress that affected leaves the most, damaging this tissue and causing up to a 360% increment in electrolyte leakage compared to the control (Table 1).

**TABLE 1:** Changes in hydrogen peroxide (H_2_O_2_, μg g^−1^ FW), malondialdehyde (MDA, μg g^−1^ FW) and Membrane Stability Index (MSI, %) in control plants and plants under single (salinity, drought, heat and cold) stresses and their combination. Data represent the mean ± standard deviation of 4 plants. Different lowercase letters indicate differences between treatments after one‐way ANOVA with Duncan's post hoc test, while asterisks indicate significant differences (* *p* < 0.05; *** *p* < 0.001).

	H_2_O_2_ (μg g^−1^ FW)	MDA (μg g^−1^ FW)	MSI (%)
**Control**	19.9 ± 2.3 d	5.0 ± 0.3 d	3.2 ± 0.2 bc
**Cold**	23.7 ± 2.1 cd	8.1 ± 0.8 c	3.1 ± 0.5 bc
**Drought**	29.1 ± 0.8 bc	8.3 ± 0.8 c	3.5 ± 1.1 bc
**Heat**	23.2 ± 0.9 cd	8.9 ± 1.2 c	3.3 ± 2.2 bc
**Salinity**	30.9 ± 2.1 b	15.9 ± 2.7 b	11.5 ± 6.5 a
**Drought × Cold**	23.6 ± 1.7 cd	8.5 ± 1.0 c	3.2 ± 1.1 bc
**Drought × Heat**	28.2 ± 5.2 bc	8.5 ± 2.3 c	2.3 ± 0.7 c
**Salinity × Cold**	37.2 ± 9.0 a	19.4 ± 2.8 a	8.8 ± 1.8 a
**Salinity × Heat**	26.7 ± 0.5 bcd	13.0 ± 3.4 b	7.2 ± 3.7 ab
**Significance**	*	***	***
**LSD**	5.8	3.3	4.6

### 3.3 Metabolomics profile of leaves

An untargeted metabolomics approach was used to investigate the effect of different stresses and their combinations on the metabolomic profile of *Arabidopsis thaliana* leaves. This approach allowed us to annotate 2124 putative compounds. The list of compounds, together with individual raw data abundance, retention time and composite mass spectra, are reported in Supplementary Table S4, whereas the raw data are published in the repository MetaboLights (Yurekten et al., 2024) under study ID MTBLS9669.

**FIGURE 2:**
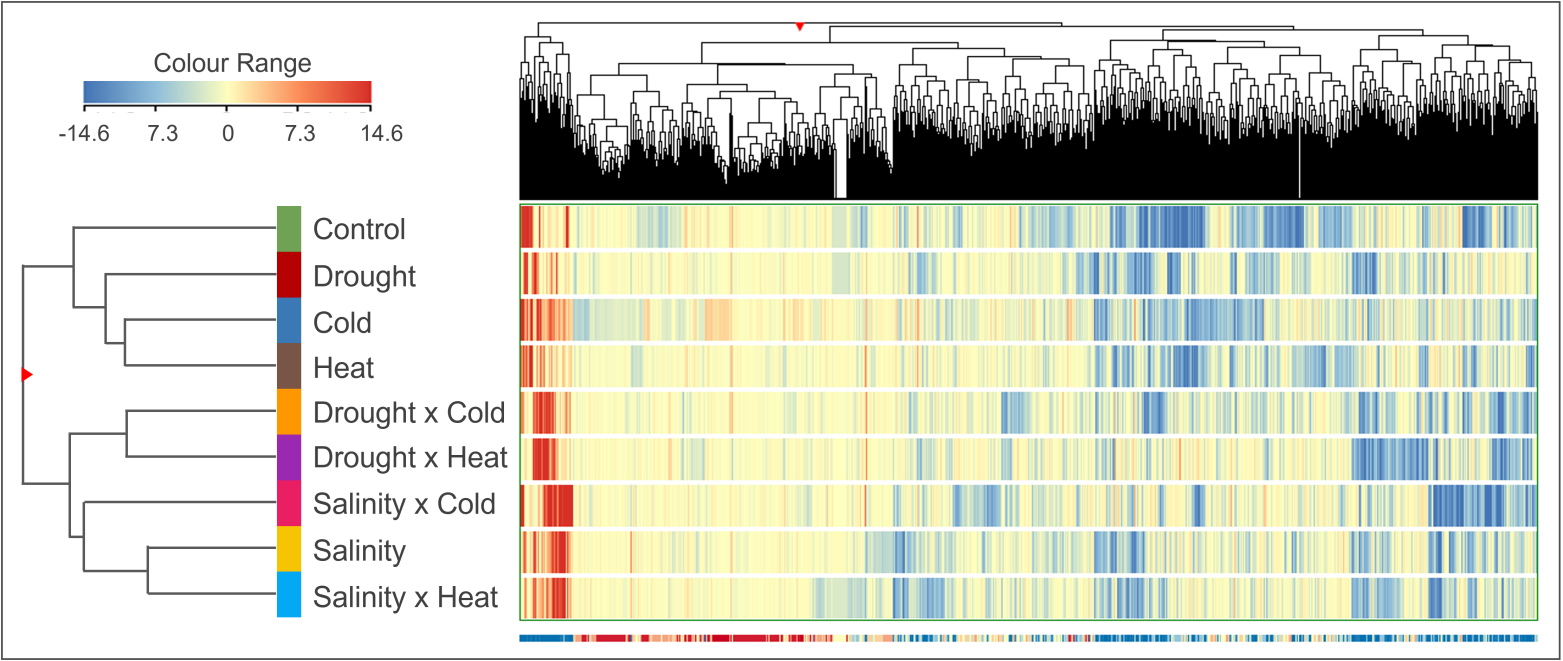
Unsupervised hierarchical clustering analysis (HCA) of the metabolomic profile of leaves obtained through UHPLC‐ESI/QTOF‐MS analysis of *Arabidopsis thaliana* grown under control, single stress conditions (e.g., cold, drought, heat, salinity) and their combination. Hierarchical clusters (linkage rule: Ward; distance matrix: Euclidean) were based on the fold‐change‐based heat map of compounds' normalized intensities.

First, unsupervised hierarchical cluster analysis was performed to investigate patterns across the conditions considered naively and to provide a hierarchical picture of the factors under study (Figure 2). As expected, the heat map based on fold‐change highlighted a distinct metabolomic profile depending on the stress applied and provided a hierarchical overview of the different conditions. Two main clusters are visible, the first including control and all single stresses except for salinity, which on opposite clustered together with all combined stresses; notably, drought and salinity‐related multiple stresses clustered distinctively.

Volcano analysis (FC >2, *p* < 0.05) highlighted 566 compounds being statistically different from the control in at least one treatment (Table S5). Specifically, for individual stresses, the number of differentially accumulated compounds (DACs) was 132 for C, 71 for H, 166 for D and 259 for S. Metabolites that differed under combined stresses were 204 for D×C, 313 for D×H, 244 for S×C and 274 for SxH. The selected compounds were then plotted on the PlantCyc pathway tool to better understand the metabolic impact driven by the different stresses applied compared to the control (Figure S1). According to the pathway tool, drought and salinity treatments and their combinations strongly mediated secondary metabolites, hormones, and amino acid biosynthesis. Among secondary metabolites, the classes showing the stronger modulation were N‐containing compounds (including glucomalcommin; S‐magnoflorine; caffeoylserotonin), S‐containing compounds (such as methiin and gamma‐L‐glutamyl‐(S)‐methyl‐L‐cysteine) and Terpene‐related compounds (such as acetoacetyl‐CoA; 10‐deoxysarpagine; phytyl diphosphate). Concerning amino acid biosynthesis, contrasting trends could be observed: while Arg, Glu, Ile, Lys, Pro and Thr were accumulated, Phe, Trp, and Tyr decreased in all conditions. Among hormones, melatonin and gibberellin A38 decreased in all considered conditions.

**FIGURE. 3:**
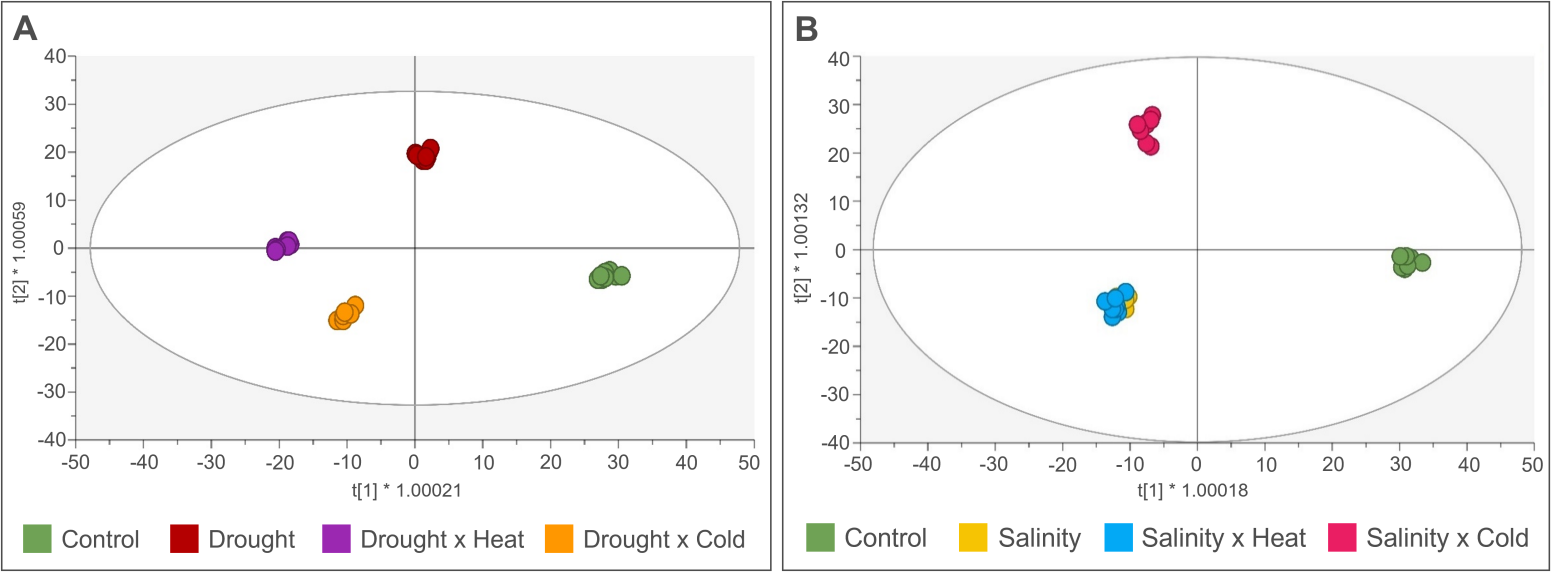
Supervised OPLS‐DA based on the metabolomic profile of Arabidopsis leaves under (A) drought condition and its combination (R2Y = 0.996; Q2Y = 0.856) and (B) salt condition and its combination (R2Y = 0.991; Q2Y = 0.831). The VIP markers (VIP‐score >1.2) extrapolated by the models are listed in Table S7 and S8, respectively.

To delve into the compounds driving this separation, two OPLS‐DA models were developed based on salinity and drought stresses and their combination (Figure 3A and B). In both cases, the models presented adequate scores (R^2^Y = 0.991 and Q^2^Y = 0.831 for S stress and its combinations and R^2^Y = 0.996 and Q^2^Y = 0.856 for D stress and its combinations). The VIP analysis (VIP score >1.2, *p* < 0.05) was then carried out, and the full list of statistically significant VIP markers under drought and salinity stresses and their combinations is provided in Tables S6 and S7, respectively. Overall, 406 compounds were found as discriminants in the OPLS‐DA model based on drought stress and its combinations. Among these, polyphenols (34), terpenes (25), alkaloids (18), indole derivates (15), fatty acids (14), glucosinolates (13) and phenylpropanoids (9) were included.

Concerning the OPLS‐DA model based on salinity stress and its combinations, 405 discriminant compounds were identified. Among these, the most represented classes were polyphenols (32), amino acid derivates (29), terpenes (28), alkaloids (19), indole derivates (14) and fatty acids (11).

**FIGURE 4:**
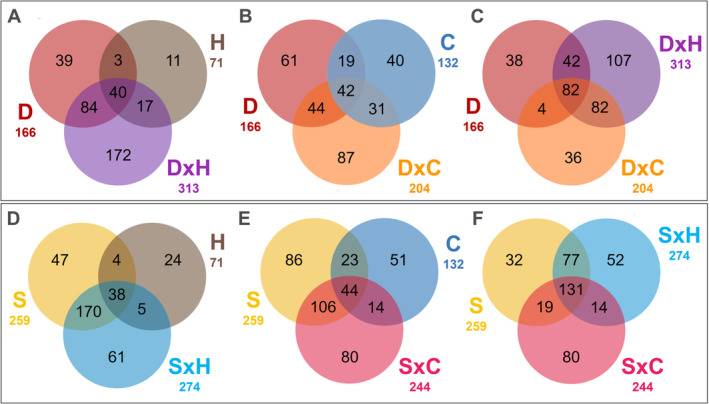
Venn diagrams comparing the differential metabolites resulting from the Volcano analysis (*p* < 0.05; FC >2) in **(A, B and C)** plants under drought condition and **(D, E and F)** under salinity stress. H=Heat; C=Cold; D=Drought; S=Salinity; D×C=Drought × Cold; D×H=Drought × Heat; S×C=Salinity × Cold; S×H=Salinity × Heat. .

To identify the metabolites in common between drought and salinity single stresses and their respective combination, Venn Diagrams were elaborated on compounds with FC >2 and *p* < 0.05 (Figure 4).

Pairwise comparisons were carried out regarding H, C, with D or S and their respective combinations (Figure 4A‐F). As already highlighted, thermal stresses applied individually modulated a smaller number of metabolites than D and S. Nonetheless, when combined with D, the dual stress effect was stronger. In more detail, 172 up to 313 compounds were specifically modulated in D × H (Figure 4A), while 87 up to 204 under D × C (Figure 4B). For drought and its combinations (D, D × C, D × H), 82 common compounds were identified (Figure 4C), 45 of which had a VIP‐score >1.2, suggesting their specific modulation under drought stress.

The analysis of the 172 DACs which specifically characterized DxH indicated an accumulation of alkaloids, flavonoids, compounds involved in the TCA cycle, sterols, and phospholipids. In particular, (S)‐magnoflorine and vincristine were the alkaloids with the highest fold change (FC). For flavonoids, the compounds with the highest variation were delphinidin‐3‐O‐(6″‐O‐malonyl)‐β‐glucoside‐3′‐O‐β‐glucoside and kaempferol‐3‐O‐α‐L‐rhamnopyranoside. Concerning the TCA cycle, 2‐(α‐hydroxyethyl)thiamine diphosphate was upregulated, a compound correlated with increased TCA cycling and carbohydrate degradation. Other upregulated compounds include ButCoA, linked to the increased regulation of phospholipids, glucodigifucoside, a sterol, and 131‐oxo‐magnesium‐protoporphyrin IX 13‐monomethyl ester, which is involved in the chlorophyll synthesis pathway. The compound γ‐L‐glutamyl‐L‐cysteine, involved in the glutathione detoxification system, was significantly downregulated. Alongside, the analysis of the 87 DACs characterizing solely D × C with respect to its single stresses indicated an accumulation of alkaloids (vincristine), sterols (glucodigifucoside), and flavonoids (malonyldaidzin). Results also suggested a down‐accumulation of γ‐L‐glutamyl‐(S)‐methyl‐L‐cysteine, a compound involved in the glutathione‐mediated detoxification system. Notably, the biosynthetic way of gibberellin A_12_ was altered due to the differential accumulation of gibberellin A_12_‐aldehyde.

Under salinity, despite the high number of shared compounds between S and its combination with H (170), and C (106), respectively. The combined effects of S × H and S × C were still differential to the respective abiotic stresses applied alone, with 61 compounds individually accumulated in S × H and 80 in S × C (Figure 4D‐E). Notably, 131 metabolites were in common across S, S × C and S × H; among them, 71 had a VIP‐score >1.2 (Figure 4F).

Compared to single S and H, DACs only under SxH indicated a mixed trend for flavonoid modulation and a down accumulation of alkaloids. Interestingly, a wide range of lipidic metabolites, such as phospholipids (1‐(18‐hydroxyoleoyl)‐*sn*‐glycrol 3‐phosphate and 1‐palmitoyl‐2‐vernoloyl‐phosphatidylcholine), and sterols ((22R,23R)‐22,23‐dihydroxy‐campest‐4‐en‐3‐one) were found to increase under this condition. Finally, the DACs characteristic only of S × C indicated an increase in alkaloids content, among which the most up accumulated were strictosidine aglicone, and (S)‐laudanosine. Alongside, despite the overall down accumulation of flavonoids, compounds such as delphinidin 3‐O‐(6″‐O‐malyl‐β‐D‐glucoside) and derivatives of myricetin and isorhamnetin were found to be more abundant compared to the control condition. Finally, the biosynthetic ways of the hormones IAA and gibberellins A_12_ were altered due to the differential accumulation of (indol‐3‐yl)acetyl‐L‐proline and (indol‐3‐yl)acetyl‐L‐aspartate and gibberellin _A12_‐aldehyde and 16,17‐dihydro‐16α,17‐dihydroxy gibberellin _A12_, respectively.

### 3.4 Impact of multiple stresses on osmolytes

A targeted approach was used to quantify key osmolytes involved in stress response, including GABA, proline, betaine, and glycine. In our experiment, the applied abiotic stresses significantly elicited proline and betaine accumulation (Table 2). Salinity and its combinations increased proline accumulation compared to the control, with S × H being the treatment with a stronger effect (14.5‐fold increase). Conversely, betaine levels were lower in D × H and D × C‐stressed plants than in control, remaining the single effect of drought stress without significant changes.

**TABLE 2:** Changes in γ‐aminobutyric acid (GABA, μg g^−1^ FW), proline (μg g^−1^ FW), betaine (ng g^−1^ FW) and glycine (μg g^−1^ FW) in control plants, and plants under single stresses (salinity, drought, heat and cold) and their combination. Data represents the mean ± standard deviation of 4 plants. Different lowercase letters indicate differences between treatments after one‐way ANOVA with Duncan's post hoc test, while asterisks indicate significant differences (n.s., non‐significant; * *p* < 0.05; *** *p* < 0.001).

	GABA μg g^−1^ FW	Proline μg g^−1^ FW	Betaine ng g^−1^ FW	Glycine μg g^−1^ FW
**Control**	4.791 ± 1.258	0.232 ± 0.034 c	2.375 ± 0.059 a	0.011 ± 0.003
**Cold**	5.161 ± 0.175	0.155 ± 0.075 c	1.950 ± 0.238 ab	0.004 ± 0.001
**Drought**	4.281 ± 0.839	0.067 ± 0.035 c	2.125 ± 0.153 a	0.006 ± 0.001
**Heat**	4.883 ± 0.474	0.179 ± 0.062 c	1.900 ± 0.346 ab	0.006 ± 0.002
**Salinity**	5.068 ± 0.468	2.556 ± 0.276 b	2.450 ± 0.370 a	0.006 ± 0.002
**Drought** × **Cold**	4.374 ± 1.048	0.091 ± 0.019 c	0.925 ± 0.287 b	0.006 ± 0.003
**Drought** × **Heat**	4.513 ± 0.332	0.188 ± 0.072 c	0.875 ± 0.377 b	0.006 ± 0.003
**Salinity** × **Cold**	5.948 ± 0.547	3.408 ± 1.393 a	2.375 ± 0.957 a	0.010 ± 0.004
**Salinity** × **Heat**	4.768 ± 0.492	2.648 ± 0.088 b	1.725 ± 0.750 ab	0.012 ± 0.011
**Significance**	n.s.	***	*	n.s.
**LSD**	1.0	0.7	1.0	6.3

### 3.5 Multifactorial ANOVA results

While one‐way ANOVA was used to point out the effect of the combination of stress factors with respect to the control rather than their interaction, a multifactorial ANOVA was carried out to investigate whether the combined stresses may result from the interaction between separate stresses. Four different two‐way ANOVA were performed to analyze the effect of each stress interaction (D×C, D×H, S×C, S×H), respectively. The *p*‐values of the single stresses and stress interactions for the photosynthetic parameters at T4, osmolytes, and biochemical assays are reported in Table 3. The exact *p*‐value results are available in Supplementary Table 8.

**TABLE 3:** The p‐value results from multifactorial two‐way ANOVA were performed on each selected stress combination. All the parameters are calculated referring to T4 that correspond to harvest. I) S, H and S × H; II) S, C and S × C; III) D, H and D × H; IV) D, C, D × C. Significance: n.s., non‐significant; * *p* < 0.05; ** *p* < 0.01; *** *p* < 0.001.

		Area	F_v_/F_m_	NPQ	qP	F_v_’/F_m_′	H_2_O_2_	MDA	MSI	GABA	Proline	Betaine	Glycine
**I**	**Heat**	n.s.	*	n.s.	n.s.	n.s.	n.s.	n.s.	n.s.	n.s.	n.s.	*	n.s.
	**Salinity**	***	***	n.s.	***	***	***	***	*	n.s.	***	n.s.	n.s.
	**Salinity** × **Heat**	n.s.	n.s.	n.s.	**	n.s.	**	*	n.s.	n.s.	n.s.	n.s.	n.s.
**II**	**Cold**	n.s.	n.s.	**	**	n.s.	n.s.	*	n.s.	n.s.	n.s.	n.s.	n.s.
	**Salinity**	***	***	**	***	***	***	***	**	n.s.	***	n.s.	n.s.
	**Salinity** × **Cold**	n.s.	n.s.	n.s.	**	n.s.	n.s.	n.s.	n.s.	n.s.	n.s.	n.s.	**
**III**	**Heat**	n.s.	***	***	***	***	n.s.	*	n.s.	n.s.	n.s.	n.s.	n.s.
	**Drought**	n.s.	n.s.	n.s.	*	n.s.	**	n.s.	n.s.	n.s.	*	n.s.	*
	**Drought** × **Heat**	n.s.	n.s.	*	*	n.s.	n.s.	*	n.s.	n.s.	**	n.s.	n.s
**IV**	**Cold**	n.s.	n.s.	***	***	*	n.s.	*	n.s.	n.s.	n.s.	n.s.	*
	**Drought**	n.s.	n.s.	**	n.s.	*	n.s.	n.s.	n.s.	n.s.	***	n.s.	n.s.
	**Drought** × **Cold**	n.s.	n.s.	n.s.	n.s.	n.s.	n.s.	n.s.	n.s.	n.s.	*	n.s.	**

In the first comparison, H had a substantial impact only on betaine accumulation and F_v_/F_m_, while S stress had a significant effect on both photosynthetic and stress‐related biochemical alterations, including projected leaves area, F_v_/F_m_, qP, F_v_′/F_m_′, H_2_O_2_, MDA, and proline. Notably, two‐way ANOVA revealed a statistically significant interaction between the effects of S and H for qP, H_2_O_2_, and MDA. Regarding the second interaction, while S stress confirmed its extensive effect on photosynthetic and biochemical parameters, C stress did not affect NPQ, qP, and MDA. Finally, the interaction between C and S turned evident for glycine and qP. In the third investigation, the severe impact of H overall photosynthetic parameters, as well as on MDA, was highlighted. Proline, glycine, H_2_O_2_, and qP were all impacted by D stress, while in dually stressed plants, the D × H interaction had a significative effect on NPQ, qP, MDA and proline. Finally, C, D, and D × C were examined in the fourth analysis. NPQ, qP, F_v_′/F_m_′, together with MDA and glycine, were all significantly impacted by C stress. Proline, F_v_′/F_m_′, NPQ, and, to a lesser extent, glycine were all impacted by D stress. Notably, D × C interaction turned significant for the proline and glycine, suggesting a key role of these osmolytes in the mechanism of the stress interaction.

## 4 DISCUSSION

### 4.1 Effect of single and combined stresses on phenotypic and photosynthetic traits

The effective evaluation of phenotypic traits and photosynthetic performance has been assessed using phenomics as a rapid and non‐invasive technique to unfold the plant's response under the selected environmental conditions. Our results show that RGB images were poorly predictive since they revealed that abiotic stresses did not affect rosette morphology in *Arabidopsis thaliana*. Different results could likely be observed in long‐term experiments. Regarding growth rate, salt‐stressed plants significantly reduced leaf area from the late phase of the treatments (T3‐T4). On the contrary, temperature stresses were applied as a single time point, thus without influencing biomass. Surprisingly, no impact on plant growth could be observed under drought conditions, probably due to the phenological phase at induction, the duration, and the severity of stress conditions. Indeed, in other experiments in *A. thaliana*, plant growth was affected when prolonged drought periods were applied, suggesting a duration‐dependent dose before the impact on biomass becomes visible. Additionally, the phenological phase during which stress is applied can be crucial in determining the overall impact, with a more pronounced effect on seedlings or young plants (Chaerle & Van Der Straeten, 2000; Harb et al., 2010; Vile et al., 2012). Overall, these results suggest that leaves surface area may not be an ideal indicator of short‐term term‐stress, even more so in multiple stress conditions.

According to previous research (Giannelli et al., 2023; Shahid et al., 2020), a reduced growth rate can correlate to lower photosynthetic performance. In fact, since the T1, corresponding to a 2‐day salinity treatment, NaCl application significantly affected the PSII efficiency, with a reduction in all photosynthetic parameters (Figure 1; Table S2). Nevertheless, the recorded values showed interesting results related to photosynthetic performance for most of the treatments. The different stresses distinguishingly impacted the non‐photochemical processes, represented by the NPQ parameter, and the photochemical efficiency, represented by the F_v_’/F_m_′ and qP traits. Specifically, while salinity affected all these parameters, possibly due to its duration, cold and heat stresses had a distinct effect on the photosynthetic apparatus, turning into an increase in the NPQ mechanism of heat dissipation in heat‐stressed plants and a decrease in the number of the PSII open centres in cold‐treated samples.

In our experiment, F_v_/F_m_ appears to be a robust parameter, being affected only under salinity stress conditions and not reflecting short stress responses (e.g., cold and heat). Interestingly, despite the 9‐day drought application, any impact on the photosynthetic apparatus could be recorded, suggesting that an overall mild stress level was reached on drought‐stressed plants, in accordance with biomass results. As already reported (Suresh et al., 2012), the suitability of Chl *a* fluorescence for assessing drought, depends on the severity and duration of the stress. Despite causing a decrease in the photosynthetic rate, mainly due to stomatal closure, mild‐to‐moderate drought stress has no direct effect on the individual metabolic reactions' capacity (Brestic et al., 1995; Cornic & Massacci, 1996; Flexas & Medrano, 2002). Notwithstanding, drought stress may worsen the effect of co‐occurring stresses (e.g., qP in DxC and NPQ in DxH samples, Table S3).

### 4.2 Metabolomics analysis unravels the plant metabolic modulation under different stress treatments

Phenotyping investigations revealed the lack of unique markers suitable for investigating stress interactions. Consequently, untargeted metabolomics was performed to delve into the effect of single and combined stress on the overall metabolism beyond hypothesis‐driven specific stress markers. The unsupervised hierarchical analyses revealed the distinctive effect of each stress, confirming the necessity to unravel the intricate responses underlying the different stress treatments. This was also confirmed by the Venn diagrams on differential metabolites under salinity or drought stress, compared to their combination with heat and cold. The results pointed out that, despite shared compounds in single and dually stressed plants, a stress‐tailored response was characteristic of each condition. In fact, to a different extent, stress combinations shared similar biochemical responses with their respective single stress while keeping their singularity in the plant metabolomic signature, thus acting as a new stressor (Mittler, 2006; Zandalinas et al., 2021). When analyzing the overall impact of stress combinations, we could outline different trends. The single application of H and C did not induce a deep modulation of the metabolism compared to the control condition, likely due to the limited duration of the stress. Nonetheless, when in combination with D, it resulted in synergistic (D × H) and partially additive effects (D × C) (Zandalinas & Mittler, 2022), even in such a time restriction. Based on this, the importance of dually stressed plant analysis is confirmed, and it particularly underlines the jeopardy of heat waves in plants growing under prolonged drought periods as in field conditions (Mittler, 2006; Mittler & Blumwald, 2010). With respect to salinity combinations with H and C, a different impact emerged compared to drought. In fact, S × H and S × C resulted in an indifferent effect as the number of compounds modulated, indicating the driving effect of salinity in modulating the metabolism under short‐term temperature stresses. Interestingly, different results on the combination of heat and salinity were found among species (e.g., synergistic in *Arabidopsis*; (Suzuki et al., 2016); and antagonistic in tomato (Rivero et al., 2014)), thus revealing the necessity to untangle the effect of these stressors in different plant species.

The analysis of the significant compounds (*p* < 0.05) with an FC >2 highlighted the main categories of metabolites affected by the treatments compared to the control. DACs under D × H, D × C, S × H, S × C conditions exhibit unique metabolic reprogramming. This is particularly significant for alkaloids, flavonoids, lipids, and hormone‐related metabolites. These findings point out a complex metabolic alteration linked to changes in cellular homeostasis modifications and stress adaptation, in agreement with the high H₂O₂ and MDA detected under combined stress conditions, especially S × C.

One of the most significant observations is the accumulation of specific alkaloids and flavonoids under both D × H and D × C conditions. (S)‐magnoflorine, an isoquinoline alkaloid with an anti‐oxidative effect against the oxidation of lipoproteins (Hung et al., 2007; Okon et al., 2020), was only accumulated in D × H, suggesting the specific modulaton under this condition.

In D × C, the up accumulation of flavonoids, such as malonyldaidzin, was prevalent. Within D × H, the main compounds were delphinidin‐3‐O‐(6”‐O‐malonyl)‐β‐glucoside‐3’‐O‐β‐glucoside and kaempferol‐3‐O‐α‐L‐rhamnopyranoside. Notably, Kaempferol‐3‐O‐α‐L‐rhamnopyranoside is one of the first flavonoids detected in *Arabidopsis thaliana* (Veit & Pauli, 1999) and its role under drought stress was studied in *Nigella sativa* (Hakeem et al., 2023). Given the well‐known antioxidant properties of flavonoids, their overaccumulation may enhance the protective response to oxidative stress induced by environmental stressors (Hernandez et al., 2004; Nakabayashi et al., 2014). Notable changes were also observed in sterols, with glucodigifucoside markedly increasing in D × H and D × C. Sterols are key components of cellular membranes, and their increased accumulation may be a response to preserve membrane integrity and fluidity, which are critical for cellular signaling and transport under stressful circumstances, including drought (Rogowska & Szakiel, 2020). The down‐accumulation of substances involved in the glutathione detoxification system, specifically γ‐L‐glutamyl‐L‐cysteine and γ‐L‐glutamyl‐(S)‐methyl‐L‐cysteine, is a remarkable discovery of D × H and DxC datasets. Reduced glutathione levels correlate with redox imbalance since glutathione is essential for detoxifying hazardous chemicals like ROS (Cheng et al., 2015; Rai et al., 2023). This decrease may indicate that the glutathione‐mediated detoxification pathway has been activated to mitigate oxidative damage susceptibility (Dorion et al., 2021). On the other hand, the simultaneous increase of flavonoids, with their established antioxidant properties, could suggest that cells may switch to other antioxidant strategies to counteract oxidative stress. Notably, despite not being induced under single stresses (D, H, or C), these mechanisms were common to drought in combination with both temperature stresses, suggesting a similar mechanism under these dual environmental threats. Nevertheless, the synergistic effect under D × H also resulted in specific mechanisms. Of particular interest is the modulation of phospholipids and chlorophyll biosynthesis compounds. The accumulation of butyryl‐CoA could be linked to membrane remodeling (Sharma et al., 2023), as phospholipids are necessary to maintain membrane integrity when stressors reduce the membrane's permeability or fluidity (Sun et al., 2022). An increase in ButCoA may suggest a higher phospholipid turnover to preserve the shape and function of membranes under stress. Alongside, the increase of chlorophyll biosynthetic intermediates, in particular of 131‐oxo‐magnesium‐protoporphyrin IX 13‐monomethyl ester, suggests that chlorophyll production may be actively controlled. This might be an adaptation to retain a high photosynthetic efficiency, ensuring that energy production via light absorption is not endangered under D × H stress (Muhammad et al., 2021).

The modulation of specific compounds under S × H condition included the up‐accumulation of some flavonoids such as kaempferol‐3‐O‐α‐L‐rhamnopyranoside, indicating that the plant requires enhanced cellular defense and is utilizing flavonoid biosynthesis to prevent oxidative damage or stabilize membranes in cells (Jan et al., 2021). On the other hand, the levels of other metabolites, such as sterols and phospholipids, were significantly reduced. Lower concentrations of (22R,23R)‐22,23‐dihydroxy‐campest‐4‐en‐3‐one sterol and of the crucial phospholipid (1‐(18‐hydroxyoleoyl)‐sn‐glycrol 3‐phosphate) suggest potential variations in the lipid composition of the membrane. These changes in membrane fluidity and signalling may compromise or alter the plant's ability to maintain homeostasis under S × H conditions, similarly to the effects observed during heat stress alone (Niu & Xiang, 2018).

The modulation of compounds under S × C indicated two opposite trends, with a decrease in flavonoid and an increase in alkaloid content. The accumulation of alkaloids indicates their role as antioxidants in maintaining cellular homeostasis under S × C stress conditions. Interestingly, strictosidine is an intermediate in the biosynthesis of monoterpene indole alkaloids, whose response helps in mitigating oxidative stress by enhancing antioxidant enzyme activities. Its expression, which has already been demonstrated to be differentially regulated under salinity and low temperature (Dutta et al., 2013), in our work was specifically modulated under their combination. The up‐accumulation of IAA‐Pro and IAA‐Asp was also reported. These indole‐3‐acetic acid amide conjugates can serve as storage, transport, excess IAA detoxification, and protection against peroxidative degradation (Woodward, 2005). Specifically, IAA‐Asp is generally associated as a catabolite of IAA, but it has also been suggested that auxin conjugates could be per se involved in abiotic stress tolerance as a stress response mechanism (Ludwig‐Müller, 2011).

Considering compounds in common under S and D applied alone and in commination, it is noteworthy the regulation of glucomalcommin, melatonin and phytyl diphosphate. Specifically, glucomalcommin is linked to the production of aliphatic glucosinolates in water stress response (Zhang et al., 2021), and in general, glucosinolates have been correlated to the aquaporins modulation (Martínez‐Ballesta et al., 2015). Melatonin, considered a novel phytohormone, is involved in plant growth and tolerance to abiotic stress by directly scavenging free radicals, enhancing ROS detoxification, and regulating the enzymatic and non‐enzymatic antioxidant systems (Zhang et al., 2020). Finally, the down accumulation of phytyl diphosphate, involved in the phythol pathway, can be related to the impact of abiotic stresses on photosynthetic activity, being related to chloroplast's structural changes (Gutbrod et al., 2019). In fact, alterations in the lipid ultrastructural composition can result in the release of large amounts of phytol due to chlorophyll degradation (Lippold et al., 2012).

### 4.3 The modulation of different stress markers under single and combined stress conditions

Maintenance of membrane stability has a key role in plant response to environmental stresses and therefore cell wall components and the proteo‐lipid fractions are subjected to a thigh control. Indeed, membrane stability depends on its lipid composition, which controls membrane fluidity (Rawat et al., 2021; Wang et al., 2020). Abiotic stresses can trigger lipid peroxidation due to increased reactive oxygen species. Accordingly, all the abiotic stresses evaluated in this experiment resulted in higher MDA content. Interestingly, salinity stress had the highest impact, probably due to the significant increment observed in hydrogen peroxide content compared to the other stresses, which correlated with MDA. Overall, the reported increase in reactive oxygen species and lipid peroxidation led to an important loss of membrane integrity in the plants exposed to stress by salinity.

Frequently, osmotic stress in plants under abiotic stress conditions is caused by ion imbalance and water deficiency. Some of the most studied effects on biophysical changes are reduction in cell turgor pressure, shrinkage of the plasma membrane, and physical alteration of the cell wall (Park et al., 2016). In response to stresses, osmolytes such as proline, glycine‐betaine and sugars are produced and accumulated as non‐toxic molecules (Chen & Murata, 2002). Osmolytes are mainly involved in regulating osmotic pressure but may also influence ABA levels and gene expression. Although the response to osmotic stress is fundamental, it impacts plant growth (Giannelli et al., 2024). Proline is one of the most important osmolytes and plays a role in stabilizing sub‐cellular structures, detoxifying ROS, buffering cellular redox potential, and stabilizing protein and protein complexes under stress conditions (Muchate et al., 2016; Sánchez et al., 2023). One of the effects of proline concerns the maintenance of photosystem II activity, as it prevents the damaging effect of reactive oxygen species on the thylakoid membrane (Alia et al., 1997). Proline biosynthesis is regulated by the activity of 2 *P5CS* genes; in *Arabidopsis*, the isoform *P5CS*1 is induced by osmotic and salt stresses (Yoshiba et al., 1995), as also confirmed exploiting mutants lacking the *P5CS1* gene (Székely et al., 2008). Our results are in accordance with previous literature and point out that the accumulation of proline is peculiar to stress conditions that include salinity; this may be explained by the need to limit photosystem II damage due to ROS species induced under stress conditions.

### 4.4 The effects of stress interactions

Multifactorial ANOVA analysis highlighted that the interaction between different stresses results in complex patterns. As pointed out by the one‐way ANOVA, S stress induced a strong modulation of the metabolism compared to D, as evidenced by the numerous parameters significantly affected under single stress conditions. On the other hand, despite the short‐time application of C and H, these temperature stresses could effectively impair, to a different extent, the photosynthetic and stress‐related plant physiology. Remarkably, by performing the different two‐way ANOVA models, the effect of H and C single stress was more visible when in combination with D. On the opposite, following the metabolomic findings, salinity appeared to be the driver in the modulation of the stress outcome under its respective combination, reasonably due to its strength with respect to H and C.

Despite the prevalent effect of S over most calculated parameters, there is an interaction effect between S and C on qP, which indicates a marked closure of reaction centres. Both C and S stresses impair the efficiency of open PSII reaction centres, as confirmed by the significant difference when applied alone, but their interaction resulted in a more robust reduction of qP, as both stresses impair the donor side (Kalaji et al., 2017). Surprisingly, glycine showed no difference under a single stress but was significant under their interaction, suggesting that these two stressors together may have a unique effect on its metabolism. It is well established that cold stress induces the accumulation of several amino acids, including glycine (Anzano et al., 2022); however, while the shortness of its application alone did not impact glycine accumulation when interacting with a second stress, glycine accumulation could be highlighted in stress interaction.

Similarly, S and H showed an interaction, leading to an intermediate effect for H_2_O_2_ and MDA and an additive effect for qP. While D alone had a limited effect on plant physiology, its interaction with H significantly affected photosynthetic and non‐photosynthetic quenching parameters, either showing a partial amelioration and deterioration of qP and NPQ, respectively. Both in D × H and D × C the interaction was significant for proline, resulting in an intermediate effect compared to single stress. While the accumulation of proline under drought is well established and discussed (Jogawat, 2019), it is noteworthy to report that its combination with temperature stress creates an intermediate regulation of this osmolyte, as already reported in (Dobra et al., 2010), indicating another mechanism of stress response such as sugar accumulation.

Overall, unravelling the impact and interaction(s) is complex under combined environmental stresses. Despite clarity's sake, the stress matrix is a powerful tool for revealing the overall effect of dual stresses (Zandalinas et al., 2021); when focusing on each single parameter, complexity arises and shows antagonistic, additive, or synergistic effects which depend on the plant tissue or biochemical process investigated (Mesa et al., 2024). Since several parameters show complex interactions, further investigation is required to thoroughly understand the underlying stress response interaction mechanisms.

## 5 CONCLUSIONS

Single and combined stresses caused a distinct metabolic signature in the model plant *Arabidopsis thaliana* (L.), affecting photosynthetic efficiency and response to oxidative imbalance, consequently altering plant growth. Salinity stress alone and in combination caused the most detrimental effects on plants, inducing a marked decline in growth by the sixth day of exposure, significantly affecting photosynthesis with increased non‐photochemical quenching (NPQ), and reducing PSII quantum efficiency. Conversely, drought, heat and cold stress did not affect growth and F_v_/F_m_. Noteworthy, a hierarchical prevalence of single and combined stresses could be highlighted, with salinity followed by drought showing stronger effects. The most pronounced effect on oxidative stress was found in plants exposed to salt stress, both alone and in combination with cold, with significant accumulation of H_2_O_2_ and MDA. Consistently, metabolomics pointed out that phytochemical signatures were significantly influenced by salinity, drought, and their combinations, with a specific impact on the production of hormones, amino acids and different classes of secondary metabolites like alkaloids, flavonoids and sterols. The accumulation of osmolytes had a role in mediating the stress response, with proline being accumulated up to 14.5 times in plants exposed to the heat and salinity combination. In contrast, betaine displayed a decrease in drought treatments combined with heat and cold. Our study demonstrated that combinations of stresses induced more complex physiological reactions than single stress, underscoring the necessity of deeply investigating the impacts of various combined stresses on plants.

## AUTHOR CONTRIBUTIONS

LL: Conceptualization; ES, MADG: Formal Analysis; ES, MADG, ACC: Investigation; ES, MADG, ACC, LL: Methodology; ES, MADG: Visualization; ES, MADG, ACC, LL: Writing–Original Draft Preparation; LL: Writing – Review & Editing.

## FUNDING INFORMATION

This research received no specific grant from any funding agency in the public, commercial or non‐profit sectors.

## Supporting information


**Supplementary Figure 1.** PlantCyc **(A)** metabolic pathway analysis and **(B)** details of secondary metabolism resulting from Volcano Plot analysis (FC >2, *p* < 0.05).

***Supplementary Table S1*.** RT, MS1 and MS/MS spectra of GABA, proline, betaine, and glycine, used for the targeted identification and quantification whit Quadrupole‐Orbitrap MS.
***Supplementary Table S2***. Morphological parameters (Leaf projected areas, Compactness and Roundness) of plants at T0‐T4.
***Supplementary Table S3***. Photosynthetic parameters (NQP, qP, Fm′/Fv’ and Fv/Fm) of plants at T0‐T4.
**Supplementary Table S4**. Putatively annotated compounds revealed with untargeted UHPLC‐ESI7QTOF‐MS analysis.
**Supplementary Table S5**. Compounds significant in at least one treatment (*p* < 0.05, Benjamini correction) loaded for PlantCyc pathway analysis.
**Supplementary Table S6**. Discriminant compounds (VIP score >1.2 and *p* < 0.05) resulting from supervised OPLS‐DA of D, DxH, DxC plants.
**Supplementary Table S7**. Discriminant compounds (VIP score >1.2 and *p* < 0.05) resulting from supervised OPLS‐DA of S, SxH, SxC plants.
**Supplementary Table S8**. *p*‐value results obtained from the different multifactorial two‐way ANOVA performed on each selected stress combinations. I) S, H and SxH; II) S, C and SxC; III) D, C and DxC; IV) D, H and DxH.

## Data Availability

The metabolomics data that support the findings of this study are openly available in METABOLIGHTS at https://www.ebi.ac.uk/metabolights/, reference number ID MTBLS9669. The morpho‐physiological data that support the findings of this study are available in the supplementary material of this article.
