# Supplementary figures and images for: Biochemical, photosynthetic and metabolomics insights of single and combined effects of salinity, heat, cold and drought in Arabidopsis
Source: Physiol Plant. 2025 Jan 16;177(1):e70062. doi: 10.1111/ppl.70062 (PMC11739553; doi:10.1111/ppl.70062)

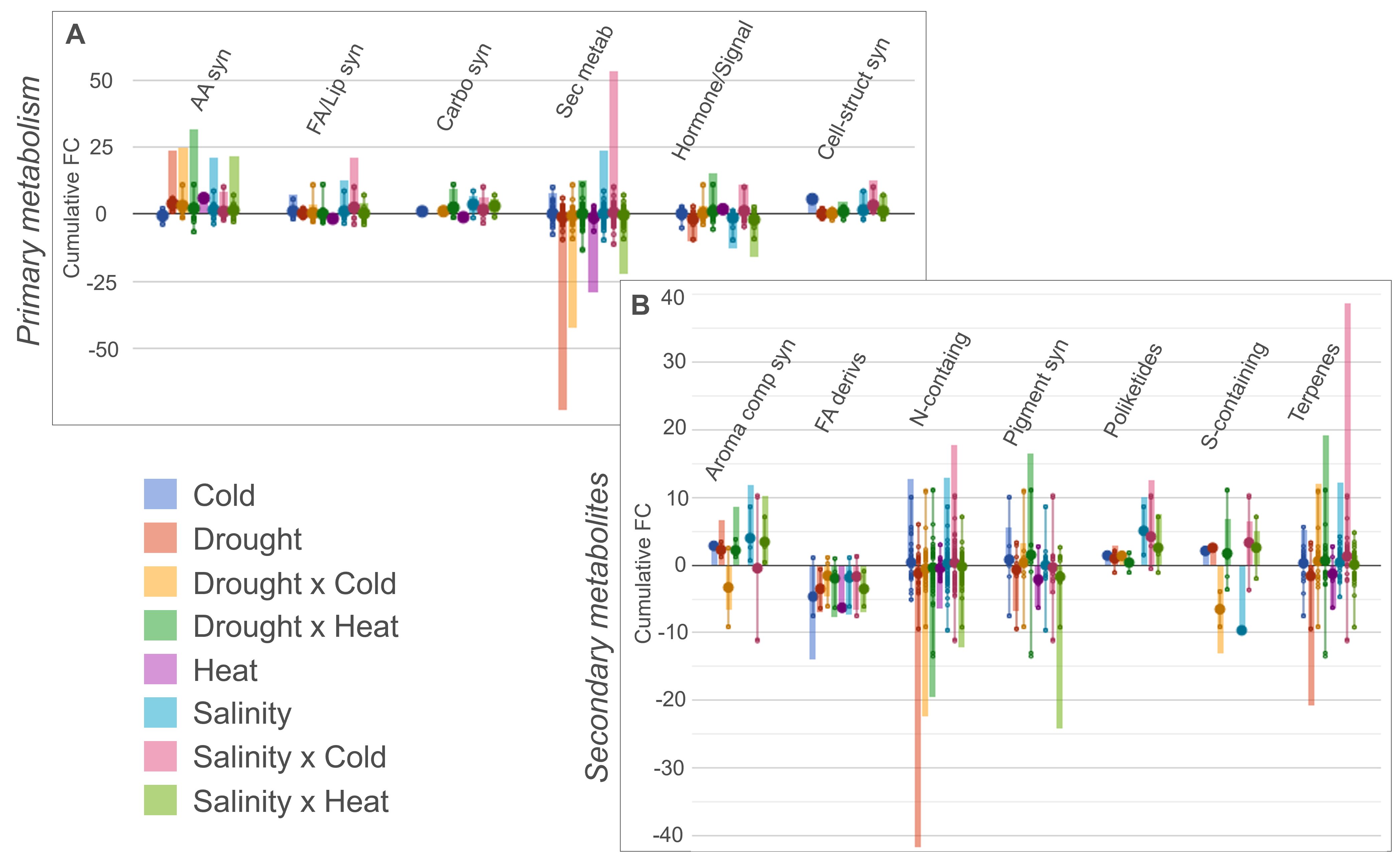

Supplement: Supplementary file 1 — Supplementary Figure 1. PlantCyc (A) metabolic pathway analysis and (B) details of secondary metabolism resulting from Volcano Plot analysis (FC >2, p < 0.05). [file PPL-177-e70062-s001.tif]
